# Psychological resilience and intention to stay among nurses: the mediating role of perceived organizational support

**DOI:** 10.3389/fpsyg.2024.1407206

**Published:** 2024-09-26

**Authors:** Jiangfeng Pu, Waner Wang, Gege Li, Zhanghao Xie, Xuanhao Fan, Ningjing Zhan, Yixuan Xu, Huigen Huang

**Affiliations:** ^1^School of Nursing, Guangdong Pharmaceutical University, Guangzhou, China; ^2^Department of Nursing, Guangdong Provincial People’s Hospital (Guangdong Academy of Medical Sciences), Southern Medical University, Guangzhou, China; ^3^School of Nursing, Jinan University, Guangzhou, China; ^4^Department of Nursing, Shantou University Medical College, Shantou, China; ^5^Department of Nursing, Southern Medical University, Guangzhou, China

**Keywords:** nurses, psychological resilience, perceived organizational support, intention to stay, the mediation effect

## Abstract

**Background:**

This study aims to analyze the relationship between psychological resilience, perceived organizational support, and intention to stay among nurses. Additionally, it explores the mediating role of perceived organizational support in the relationship between psychological resilience and nurses’ intention to stay.

**Methods:**

A cross-sectional survey was conducted between August and September 2023, involving 1,402 nurses from five Grade 3A hospitals in Guangdong. The survey utilized several instruments, including the General Information Questionnaire (GIQ), the Chinese version of the Connor-Davidson Resilience Scale (CD-RISC), the Chinese version of the Perceived Organizational Support Scale (POSS), and the Chinese version of the Intention to Stay Scale (ITSS). The obtained data were analyzed using descriptive statistics and Pearson’s correlation coefficient, while the mediating effect of perceived organizational support was assessed using the PROCESS macro mediation model in SPSS.

**Results:**

The overall mean score for psychological resilience among nurses in the five Grade 3A hospitals in Guangdong was 60.54 ± 19.17, the overall mean score for perceived organizational support was 45.77 ± 11.49, and the mean score for intention to stay was 20.82 ± 4.65. The results of the statistical analysis revealed positive correlations between psychological resilience and intention to stay (*r* = 0.388, *p* < 0.01), between perceived organizational support and psychological resilience (*r* = 0.570, p < 0.01), and between perceived organizational support and intention to stay (*r* = 0.550, *p* < 0.01). Additionally, perceived organizational support was found to mediate the relationship between psychological resilience and intention to stay, with a mediation effect value of 0.067, accounting for 71.28% of the total effect.

**Conclusion:**

Psychological resilience of nursing staff directly impacts their intention to stay and indirectly influences their caring behaviors, with perceived organizational support serving as a key mediator in both relationships. Therefore, nursing managers should implement targeted interventions to enhance nurses’ psychological resilience and perceived organizational support. Strengthening these factors can significantly increase nurses’ intention to stay in their jobs, improve the quality of care, and contribute to building a strong and stable nursing workforce.

## 1 Introduction

High nurse turnover is a critical issue in global health organizations, negatively impacting the stability of the nursing workforce, the quality of nursing services, and the cost of nursing manpower mobility (Roche et al., 2015).

The WHO Global Strategy on Human Resources for Health: 2030 Health Workforce study predicts a global nursing shortage of up to 7.6 million (World Health Organization, 2010).

As opposed to expanding the number of nursing graduates, retaining the existing workforce is widely acknowledged as a critical solution to the nursing shortage. Intention to stay is a strong predictor of nurse retention (Efendi et al., 2019; The International Center for Nurse Migration, 2023).

Poor nurse retention not only negatively affects patient health outcomes but also leads to increased workload and higher stress levels among nurses.

Ultimately, this results in a decline in the quality of care provided, endangering patient security and increasing mortality rates (Varasteh et al., 2022; Valizadeh et al., 2016).

The Job Demands-Resources (JD-R) theory (Bakker and Demerouti, 2007) posits that job resources–physical, psychological, social, or organizational aspects of a job–serve to motivate employees, help them achieve work goals, manage job demands, and promote learning and personal growth.

Personal resources and job resources share a reciprocal relationship. Resilience, as a personal resource, and organizational support, as a job resource, interact to enhance the overall job resources, which in turn improves work performance (Bakker et al., 2023).

Psychological resilience, defined as the ability to self-adjust and adapt in the face of adversity and stress, helps individuals manage workplace challenges and achieve personal growth (Troy et al., 2023).

Nurses in China often experience high levels of psychological stress and heavy clinical workloads, and their intention to stay in the field is influenced by their psychological resilience (Huang et al., 2020).

The majority of Chinese nurses have low levels of psychological resilience, leaving room for improvement (Chen et al., 2020).

Research by Gensimore et al. (2020) demonstrated that nurses with higher levels of psychological resilience experience less burnout and greater work engagement. Stabilizing the nursing workforce and enhancing care quality can be achieved by strengthening the psychological resilience of nursing staff (Liu et al., 2021).

Therefore, higher levels of psychological resilience help nurses respond positively to workplace stress and challenges, handle emergencies more calmly and effectively, and maintain and enhance their occupational health (Yuan et al., 2022).

The term “perceived organizational support” refers to employees’ perception of the support they receive from their employer, including the extent to which they feel valued for their contributions and the degree to which they believe the organization is attentive to their well-being (Eisenberger et al., 1986).

An essential component of nurses’ professional experiences is perceived organizational support, which is a strong predictor of their intention to remain in their current positions (Li et al., 2020).

High levels of perceived organizational support increase organizational commitment, foster a sense of organizational duty, reduce burnout, and improve retention (Cheng et al., 2020). Furthermore, studies have demonstrated a favorable association between nurses’ perceived organizational support and psychological resilience (Xue, 2023).

Nurses with high levels of mental toughness are better equipped to cope with work pressure, remain flexible and resilient in the face of challenges, and are more likely to receive hospital recognition and support, which in turn enhances their career development, creating a mutually reinforcing and positive relationship (Yuan et al., 2022).

**Figure 1:**
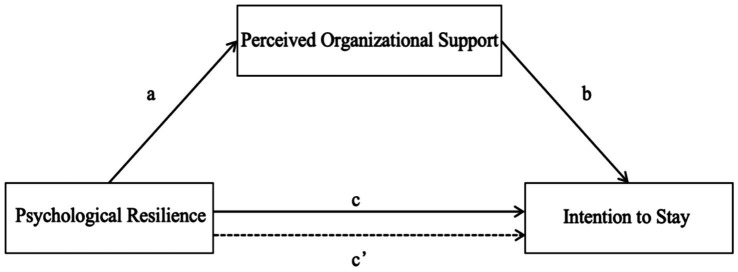
The theoretical model.

Although previous research has demonstrated a correlation between nurses’ psychological resilience, perceived organizational support, and their intention to stay (Xue, 2023; Huang et al., 2016; Lee et al., 2023), no mediation analysis has examined the relationship between these factors. Therefore, this study is based on the JD-R model, with psychological resilience as a personal resource, organizational support as a job resource, and intention to stay as work performance, to explore the relationship between the three. We suggest the following model conceptual diagram based on the aforementioned information, as shown in Figure 1.

This research aims to thoroughly explore the impact mechanisms of psychological resilience and perceived organizational support on nurses’ intention to stay. The goal was to provide a theoretical foundation for managers to better understand the psychological needs of nurses, improve their retention intentions, and promote the implementation of quality healthcare service measures.

We propose the following hypotheses:

*H1:* Psychological resilience is positively correlated with intention to stay (path c).

*H2:* Psychological resilience is positively correlated with perceived organizational support (path a).

*H3:* Perceived organizational support is positively correlated with intention to stay (path b).

*H4:* Perceived organizational support mediates the effect of psychological resilience and intention to stay (path c’).

## 2 Materials and methods

### 2.1 Study design and participants

Between October and December 2023, a cross-sectional study was conducted in Guangdong Province, China, employing snowball and convenience sampling methods. The sample size was determined according to Wu (2010) recommendation that a sample size exceeding 200 is necessary to establish a robust model. Thus, our objective was to gather a minimum of 200 valid questionnaires. The inclusion criteria are as follows: (Roche et al., 2015) possession of a nursing practice certificate and participation in clinical work; (World Health Organization, 2010) self-assessment of no mental or cognitive impairment; and (Efendi et al., 2019) volunteering to participate in this study. The exclusion criteria are as follows: (Roche et al., 2015) practice nurses and trainee nurses who have not obtained the practice qualification and (World Health Organization, 2010) nurses who did not work in a hospital during the survey period (such as studying abroad, not participating in clinical work while engaged in management tasks, and those who requested leave or were hospitalized).

### 2.2 Data collection

In this study, a QR code and a link were generated using the questionnaire network. The researcher then shared the link and QR code with hospital nursing department directors through the WeChat platform. These directors coordinated the participation of nurses who met the inclusion criteria. Nurses had the option to participate voluntarily and could stop at any point, whether they answered all, some, or none of the questions, without fear of being penalized. To avoid duplication, each account and IP address was restricted to a single submission.

### 2.3 Measurements

#### 2.3.1 General information questionnaire

The General Information Questionnaire, designed by the research team, elicited information on sex, age, marital status, level of education, professional title, years of service, department, employment status, and reasons for choosing a nursing profession.

#### 2.3.2 Chinese version of the Connor-Davidson resilience scale

The Connor-Davidson Resilience Scale (CD-RISC), developed by Connor and Davidson (2003), has been validated in its Chinese version by Yu and Zhang (2007). This scale comprises 25 items and assesses three dimensions of resilience: strength, optimism, and overall resilience. Strength refers to an individual’s ability to draw on inner resources when responding to stress, while optimism involves maintaining a positive outlook on the future. Resilience refers to an individual’s perseverance and strong will in facing challenges and adversity. Responses are measured on a 5-point Likert scale ranging from 0 (never) to 4 (always), resulting in a total score range of 0 to 100. Higher scores indicate greater levels of psychological resilience among nurses. The Chinese version of the scale demonstrated strong internal consistency, with Cronbach’s *α* coefficient of 0.91 for the total score and 0.88, 0.80, and 0.60 for the three subscales. In the current study, Cronbach’s *α* coefficient was calculated to be 0.976.

#### 2.3.3 The Chinese version of the perceived organizational support scale

The Chinese version of the Perceived Organizational Support Scale (POSS) was revised by Zuo and Yang (2009) according to the characteristics of the nursing profession. The scale consists of 13 items, categorized into two dimensions: emotional support and instrumental support. Items 1 to 10 pertain to emotional support, which involves the organization providing employees with encouragement and emotional backing. Items 11 to 13 address instrumental support, which refers to the organization’s provision of necessary tools and resources to perform their work efficiently. The scale uses a 5-point Likert scale, ranging from 1 (strongly disagree) to 5 (strongly agree), with total scores ranging from 13 to 65. Higher scores indicate stronger perceived organizational support among nurses. In this study, Cronbach’s *α* coefficient was 0.983, indicating high reliability.

#### 2.3.4 The Chinese version of the intention to stay scale

Tao Hong translated and revised the intention to stay scale (ITSS) into Chinese (Tao and Wang, 2010). The questionnaire consists of 6 entries, using a Likert 5 scale, with scores of 1 (unlikely) to 5 (very likely), of which entries 2, 3, and 6 are reverse scored, with a total score of 6 to 30, with higher scores indicating a greater willingness to stay in the job. The questionnaire has now been applied to groups such as nurses and nursing students, and Cronbach’s α coefficient in the current study was 0.786.

### 2.4 Statistical analyses

IBM SPSS 27.0 was used for analysis. First, the data with the normal distribution of continuous variables were represented by mean and standard deviation. The categorical variables were described as frequency and percentage. Second, Pearson’s correlation analysis was used to analyze the relationship between psychological resilience, perceived organizational support, and intention to stay for normally distributed data. Finally, we used the PROCESS macro mediation model (model 4) (Hayes, 2013) in SPSS. We set psychological resilience as the independent variable, perceived organizational support as the intermediary variable, and intention to stay as the dependent variable to estimate the size and significance of the mediation effect. The bootstrap method was used, with a sample size of 5,000 and a confidence interval of 95%. The mediation effect was considered significant if the confidence interval did not include zero (Zhang et al., 2021).

### 2.5 Ethical considerations

The study protocol was ethically approved by the Ethics Committee of Guangdong Provincial People’s Hospital (Approval No. KY2023-433-03). Before the start of the study, all participants were provided with detailed information about the purpose and procedures of the study. Subsequently, they signed an informed consent form, indicating their voluntary participation and understanding that their responses would remain anonymous. The researchers implemented measures throughout the data collection and analysis process to ensure the confidentiality of the data and to prevent any unauthorized use of the data.

## 3 Results

### 3.1 Descriptive statistics

The sample consisted of 1,402 nurses from five Grade 3A hospitals in Guangdong Province. Of these, 57 were male individuals, representing 4.1% of the sample, while 1,345 were female individuals, accounting for 95.9% of the total number of nurses. In terms of education level, 455 nurses (32.5%) held a junior college degree, 937 nurses (66.8%) had undergraduate degrees, and 10 nurses (0.7%) had a master’s degree or higher. Table 1 shows the remaining demographic features of this sample.

**Table 1:** Demographic characteristics of participants(*N* = 1,402).

Variables	Categories	*N*	%
Sex	Male	57	4.1
	Female	1,345	95.9
Age (in years)	≤30	646	46.1
	31 ~ 40	489	34.9
	≥41	267	19.0
Marital status	Unmarried	423	30.2
	Married	959	68.4
	Divorce or widow	20	1.4
Education level	Junior college	455	32.5
	Undergraduate	937	66.8
	Master’s degree or above	10	0.7
Professional title	Junior	916	65.3
	Intermediate	390	27.8
	Senior	96	6.8
Years of service (in years)	≤5	273	19.5
	6 ~ 10	478	34.1
	11 ~ 20	401	28.6
	≥21	250	17.8
Department	Internal Medicine	486	34.7
	Surgery	258	18.4
	Obstetrics or gynecology	106	7.6
	Pediatrics	74	5.3
	Intensive care unit	187	13.3
	Medical technology department	67	4.8
	Others	224	16.0
Employment status	Permanent	545	38.9
	Temporary	857	61.1
Reasons for choosing the nursing profession	Interests	319	22.8
	Parent or teacher recommendation	875	62.4
	Others	208	14.8

### 3.2 Current situation of psychological resilience, perceived organizational support, and intention to stay among nurses

Table 2 shows the scores of psychological resilience, perceived organizational support, and intention to stay among nurses. The overall mean scores for resilience, perceived organizational support, and intention to stay are 60.54 ± 19.17, 45.77 ± 11.49, and 20.82 ± 4.65, respectively.

**Table 2:** Psychological resilience, perceived organizational support, and intention to stay scores of nursing staff (*N* = 1,402, $\bar{x}\pm s$).

Variables	Items	Scoring range	Mean ± SD
Psychological resilience	25	0 ~ 100	60.54 ± 19.17
Tenacity	13	0 ~ 52	30.31 ± 10.44
Strength	8	0 ~ 32	20.45 ± 6.34
Optimism	4	0 ~ 16	9.78 ± 3.20
Perceived organizational support	13	13 ~ 65	45.77 ± 11.49
Emotional support	10	10 ~ 50	34.81 ± 9.05
Instrumental support	3	3 ~ 15	10.96 ± 2.67
Intention to stay	6	6 ~ 30	20.82 ± 4.65

### 3.3 Correlation analysis of psychological resilience, perceived organizational support, and intention to stay of nursing staff

Table 3 shows the results of the correlation analysis between the total psychological resilience score and dimensions, the total perceived organizational support score and dimensions, and the willingness of nursing staff to stay in their jobs. Intention to stay was significantly and positively correlated with psychological resilience (*r* = 0.388, *p* < 0.01) and perceived organizational support (r = 0.550, *p* < 0.01); psychological resilience was significantly and positively correlated with perceived organizational support (*r* = 0.570, *p* < 0.01).

**Table 3:** Correlation analysis between psychological resilience, perceived organizational support, and intention to stay of nursing staff (*N* = 1,402).

Variables	Tenacity	Strength	Optimism	Psychological resilience	Emotional support	Instrumental support	Perceived organizational support	Intention to stay
Tenacity	1.000							
Strength	0.902^*^	1.000						
Optimism	0.799^*^	0.865^*^	1.000					
Psychological resilience	0.976^*^	0.967^*^	0.889^*^	1.000				
Emotional support	0.545^*^	0.566^*^	0.487^*^	0.565^*^	1.000			
Instrumental support	0.508^*^	0.556^*^	0.474^*^	0.540^*^	0.892^*^	1.000		
Perceived organizational support	0.547^*^	0.574^*^	0.494^*^	0.570^*^	0.994^*^	0.935^*^	1.000	
Intention to stay	0.338^*^	0.439^*^	0.349^*^	0.388^*^	0.541^*^	0.536^*^	0.550^*^	1.000

*p < 0.01.

### 3.4 Analysis of the mediating effect of perceived organizational support between psychological resilience and intention to stay

The results showed that the 95% CI for both the direct and indirect effects of psychological resilience on nurses’ intention to stay in the profession did not include zero, suggesting that perceived organizational support partially mediates the relationship between psychological resilience and intention to stay.

The direct effect of psychological resilience on intention to stay was 0.027, the mediating effect was 0.067, and the total effect was 0.094, with the mediating effect percentage accounting for 71.28% of the total effect (see Table 4).

**Table 4:** Effect values of psychological resilience, perceived organizational support, and intention to stay of nursing staff from five Grade 3A hospitals in Guangdong Province.

Effect	Path	B	SE	P	95%*CI*	Percent (%)
Direct effect	CD-RISC→ITSS	0.027	0.007	<0.001	0.014 ~ 0.040	28.72
Indirect effect	CD-RISC→POSS→ITSS	0.067	0.005	<0.001	0.057 ~ 0.078	71.28
Total effect		0.094	0.006	<0.001	0.082 ~ 0.106	

## 4 Discussion

### 4.1 Analysis of the current situation of nursing psychological resilience, perceived organizational support, and intention to stay

The results of this study initially showed that the psychological resilience score of nurses was 60.54 ± 19.17, which is considered a medium level. This score was lower than that in the Han Binru study which included a psychological resilience survey of 71,477 nurses in China (Han et al., 2021). This finding could be attributed to the fact that the study’s participants were from Guangdong Province in China, a region that is more economically developed.

Due to the nature of their work, patient interactions, workplace conditions, and societal expectations, nurses experience higher levels of psychological stress and work pressure compared to other professions (Chen et al., 2024). In China, the rapid advancement of the nursing profession, heavy workload, frequent occurrence of nurse–patient disputes, and the psychological pain brought about by the discrepancy between intrinsic expectations and reality have resulted in increased work pressure on nurses, causing long-term mental stress, which hinders the development of their robust psychological resilience (Pan et al., 2019).

Therefore, to help nurses express their thoughts, develop their psychological stamina, enhance their ability for psychological recovery, and actively shape and continuously strengthen their psychological resilience to deal with a variety of unexpected medical events and a constantly changing work environment, managers could arrange a variety of psychological training courses or workshops and seek advice from psychologists.

Furthermore, the results of this study showed that the perceived organizational support score among nurses was 45.77 ± 11.49, which is moderate but lower than that in Chen et al. (2023) study. In this group, 65.3% of nurses held junior professional titles. Studies have shown that nurses with higher professional titles tend to experience better job stability, adapt more easily to their work environment, and perceive greater organizational support (Wang and Wang, 2021).

Additionally, the instrumental support score of the nurses in this group was higher than the emotional support score, indicating that the organization places more emphasis on providing material support, such as welfare benefits, training opportunities, and improved working environment, while neglecting emotional care, such as psychological counseling and humanistic support.

Consequently, it is advised that managers foster a supportive organizational culture in clinical nursing practice and prioritize the beneficial effects of organizational support on nurses’ work. By implementing initiatives such as humanizing the scheduling process, offering frequent skill-building opportunities, and encouraging further education, managers can enhance nurses’ perceptions of organizational support and create an environment that promotes professional growth. In addition, proactive attention should be paid to nurses’ emotional well-being by providing emotional support. This can help alleviate negative emotional states, prevent burnout, and ultimately improve job satisfaction.

Subsequently, the results of this study showed that the total score of nurses’ intention to stay in their jobs was 20.82 ± 4.65, which is considered a medium level. This finding is similar to the results of the studies conducted by Wang and Wang (2021) and Zhang et al. (2019) and slightly lower than the results of the study conducted by Hao et al. (2022). The highest score (4.04 ± 1.11) was given to the reverse-scored item 3 (How often do you try to find a new job?). The lowest score (2.68 ± 1.19) was given to the reverse-scored item 2 (Would you consider leaving your current nursing job position if you had another job opportunity?) in the Intention to Stay Scale, which indicates that nurses do not actively search for another job with a high frequency but are willing to give up their current nursing job position. Upon analyzing the reasons, it is notable that the respondents of this study come from Guangdong Province, a region well-known for abundant medical resources and robust economic development, offering more opportunities for employment and promotion (Gan et al., 2020). The promising prospect of re-employment after leaving the job encourages nurses to aspire for better jobs. Furthermore, all the nurses investigated in this study work in public hospitals, and even though the hospitals provide a certain degree of remuneration and benefits, the uncertainty of the job market in the nursing profession and the limitations of job searching compel the nurses to remain in their current nursing positions. It can be seen that the nurses’ intention to stay in their jobs has not yet been stabilized. This suggests that in order to foster and preserve on-the-job nursing talent and stabilize the nursing workforce, nursing administrators must consider the unique demands of nurses and develop effective career advancement programs tailored to these professionals.

### 4.2 Correlation analysis of nursing psychological resilience, perceived organizational support, and intention to stay

The results of this study showed a positive correlation between psychological resilience and intention to stay, validating the H1 hypothesis that higher levels of psychological resilience increase the willingness to stay, while insufficient resilience leads to higher turnover rates. This result aligns with the findings of Lee et al. (2023), which showed that nurses with greater psychological resilience are better equipped to adapt to their work environment, increasing their sense of commitment and intention to stay. Similarly, Wang et al. (2021) study revealed that psychological resilience allows nurses to quickly adapt to difficult situations, effectively manages stress and challenges, and positively influences retention intentions.

Based on these findings, it is recommended that managers implement psychological resilience training and support measures, such as mental health resources, stress management training, and team-building activities. These interventions can enhance psychological resilience of nurses through structured intervention strategies, in order to provide a work environment that supports nurses’ career development and improves their intention to stay in their jobs, which helps in creating a more stable and efficient nursing workforce.

The positive correlation between nurses’ perceived organizational support and psychological resilience verified hypothesis H2, which posits that higher levels of organizational support lead to increased psychological resilience. This finding is consistent with the results of Xue (2023) research, indicating that organizational support can enhance nurses’ self-awareness, self-confidence, teamwork, and communication, thereby improving their psychological resilience. Therefore, nursing managers should prioritize building strong organizational support systems, improving the work environment, and providing resources that promote nurses’ mental health and career development. The positive correlation between nurses’ perceived organizational support and retention intention validates hypothesis H3, which posits that increasing nurses’ perceived organizational support promotes their retention intention, which is consistent with the study of Huang et al. (2016). A high level of perceived organizational support can stimulate nurses’ sense of responsibility, increase organizational commitment, reduce burnout, and thus increase retention intentions (Bobbio et al., 2012). Nursing work is characterized by high risk and high workload, and to cope with this stress, nurses need to obtain a full range of support from their families, organizations, and society. Organizational support is crucial for long-term nursing retention. When nurses perceive care and support from their organization, they are more likely to develop stable working relationships and remain in their positions (Li et al., 2020). This indicates that nursing managers have the potential to enhance nurses’ perception of organizational support through initiatives such as vocational training, enhancements to the work environment, and the implementation of effective communication channels. Moreover, fostering a supportive organizational culture can play a pivotal role in bolstering nurses’ commitment to remaining in their roles.

### 4.3 Analysis of the mediating effect of perceived organizational support between psychological resilience and intention to stay in nurses

Finally, the results of this study showed that nurses’ perceived organizational support partially mediated the relationship between psychological resilience and intention to stay, with a mediation effect value of 0.067, which accounted for 71.28% of the total effect. This finding suggests that nurses’ psychological resilience directly predicts their retention intentions and indirectly influences these intentions through their perceived organizational support, validating hypothesis H4. Perceived organizational support is employees’ overall perception and belief about how the organization perceives their contributions and cares about their interests. Nurses with high psychological resilience are more capable of coping with work challenges. Nurses who are more flexible and resilient in dealing with stress, complexity, and change at work are more likely to be recognized and supported by the organization, thereby enhancing nurses’ sense of support for the organization and establishing a mutually reinforcing relationship. When individuals genuinely perceive the organization’s care and support during their work, it enhances nurses’ trust, job satisfaction, and commitment, ultimately increasing their willingness to remain with the organization (Li et al., 2020). The perceived organizational support mediated the relationship between nurses’ retention intentions to some extent. This process reveals the strength of the support, further confirming the JD-R model.

Hence, nursing managers are encouraged to recognize the significance of nurses’ psychological resilience and perceived organizational support in influencing their intention to remain in their roles. This involves two key strategies: first, prioritizing nurses’ mental well-being by implementing proactive measures to cultivate psychological resilience. This could include developing tailored resilience-building programs that help nurses navigate the complexities of their roles within the dynamic healthcare landscape. Second, this can be done by creating structured and equitable career advancement pathways, enhancing career development opportunities for nursing personnel, and accommodating flexible scheduling preferences to foster a supportive workplace environment that aligns with nurses’ needs.

## 5 Limitations

This study has limitations, such as a small sample size. In addition, due to regional differences, this study’s results cannot represent nurses’ overall situation in China. In future studies, the sampling range could be expanded, and the relationship between psychological resilience, perceived organizational support, and intention to stay could be further explored among nurses in different regions and at different levels of intention. Attention could also be paid to other variables that may affect nurses’ intentions to stay in their jobs.

## 6 Conclusion

In conclusion, psychological resilience has a direct and indirect predictive effect on retention intentions. Perceived organizational support plays a mediating role in psychological resilience and retention intentions. Nursing managers should pay attention to the interrelationships and roles of psychological resilience, perceived organizational support, and retention intentions. They should adopt targeted interventions to enhance nurses’ psychological resilience and perceived organizational support, thereby improving their retention intentions.

## Data Availability

The original contributions presented in the study are included in the article/supplementary material, further inquiries can be directed to the corresponding author.
